# PKR deficiency delays vascular aging via inhibiting GSDMD-mediated endothelial cell hyperactivation

**DOI:** 10.1016/j.isci.2022.105909

**Published:** 2022-12-30

**Authors:** Zhouyangfan Peng, Xiqing Tan, Liangpeng Xie, Ze Li, Sufang Zhou, Yapei Li

**Affiliations:** 1 Department of Health Management Center, the Third Xiangya Hospital, Central South University, Changsha, China; 2 Department of General Practice, the Third Xiangya Hospital, Central South University, Changsha, China; 3 Department of Hematology and Critical Care Medicine, the Third Xiangya Hospital, Central South University, Changsha, China; 4 School of Basic Medical Sciences, Guangxi Medical University, Nanning, China

**Keywords:** Biological sciences, Physiology, Cellular physiology, Cell biology

## Abstract

Vascular aging is an independent risk factor for cardiovascular diseases, but the regulatory mechanism is not clearly understood. In this study, we found that endothelial PKR activity is elevated in aging aorta tissues, which is accompanied with increased endothelial cell hyperactivation, IL-1β and HMGB1 release and vascular smooth muscle cell (VSMC) phenotype transforming. Global knockout of PKR exhibits significantly delayed vascular aging compared to wild-type mice at the same age. *In vitro*, using PKR siRNA or the cell hyperactivation inhibitor glycine or disulfiram can effectively inhibit H_2_O_2_ or palmitic acid-induced endothelial cell hyperactivation, IL-1β and HMGB1 release and co-cultured VSMC phenotype transforming. These results demonstrate that endothelial PKR activation induces GSDMD-mediated endothelial cell hyperactivation to release HMGB1 and IL-1β, which promotes the phenotype transforming of VSMC and subsequent accelerates the process of vascular aging. These discoveries will help to explore the new drug target to inhibit vascular aging.

## Highlights

• PKR deficiency delays vascular aging
• Endothelial PKR deficiency inhibits GSDMD-mediated endothelial cell hyperactivation
• Endothelial cell hyperactivation triggers VSMCs phenotype transformation

## Graphical abstract

**Figure:**
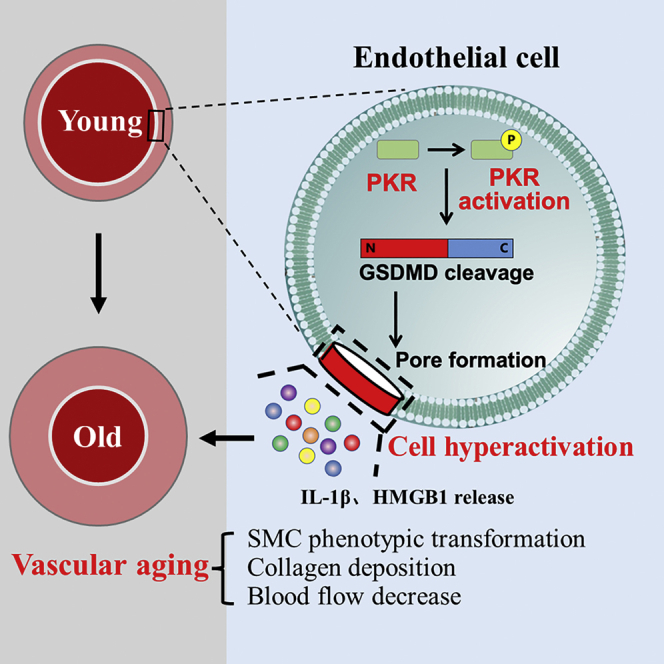

## Introduction

The population aging is an important economic and societal problem around the world.^1^ Cardiovascular diseases (CVDs) such as hypertension, atherosclerosis, and stroke are the most common causes of death among elderly people worldwide.^2^ According to the cardiovascular epidemiological statistics, the incidence of CVDs among people aged 25–44 is only 5.5%, but among people over 65, the incidence of CVDs is as high as 41%.^3^ Moreover, more than 90% of patients with CVDs are over 40 years old.^4^ Vascular aging, as one of the main physiological characteristics of aging, is regarded as an independent risk factor for CVDs.^5^ With an increase in age, arterials gradually undergo a series of functional and structural alterations, including endothelial cell dysfunction, smooth muscle cell proliferation, and collagen deposition, which lead to an increase of vascular stiffness and decrease of blood flow.^3^^,^^6^ However, our ability to slow or reverse the progression of vascular aging is limited because of our poor understanding of mechanisms that control this process. Therefore, it is of great significance to explore the molecular mechanism of vascular aging to prevent the process of age-related CVDs.

Double-stranded RNA-dependent protein kinase R (PKR) as a serine/threonine protein kinase can induce autophosphorylation at Thr 446 and Thr 451 sites by specifically binding to stimulus response elements, which plays an important role in regulating various signal pathways of innate immune diseases.^7^ In the past, it was considered that PKR could only be activated by infectious agents, TLRs ligands, cytokines and other inherent immune-related factors, to regulate the activation of immune signal pathways and release of immune inflammatory factors.^8^ In our recent studies, PKR has been revealed to be a key target in promoting endothelial cell senescence and pulmonary hypertension (PH) mediated endothelial injury.^9^^,^^10^ In normal physiological conditions, the endothelium is a crucial regulator of vascular physiology and produces several substances to protect the layer of arteries. However, injured endothelial cells become the initial contributor to promote the development of cardiovascular diseases in a pathological phenotype.^11^ We previously reported that PKR triggered IL-1β and HMGB1 release to induce PH development, although how endothelial PKR promotes IL-1β and HMGB1 release in vascular aging still need to be further investigated.

Gasdermin family member GSDMD has long been defined as the executor of pyroptosis, a novel programmed cell death. The N-terminal of GSDMD acts as the active fragment of GSDMD, mediates the pore-formation on cellular membrane to trigger cell membrane lysis-mediated cell death and inflammatory factors leak.^12^ However, the latest studies have found that GSDMD mediated pore-formation can also induce inflammatory factors release in living macrophage cell, which is called cell hyperactivation.^13^ In this cell state, activated GSDMD do not trigger cell death but only mediates the release of inflammatory factors and metal ion. Hence, we try to in-depth evaluate whether PKR mediated inflammatory factors release is because of mediating endothelial cell hyperactivation. Despite this, how endothelial PKR-mediated inflammatory factors release induces vascular smooth muscle cells (VSMCs) senescence is still unknown. As the main cell type within the vasculature, VSMCs are responsible for maintaining vascular homeostasis. It can present as contraction phenotype and secretory phenotype.^14^ The phenotype transforming of VSMCs from contraction phenotype to secretory phenotype is a remarkable symbol of vascular aging.^2^ In normal blood vessel, contraction phenotype VSMCs is the vast majority type of VSMCs. During the aging process, VSMCs gradually lose the contractile phenotype and acquire the proliferative and secretory phenotype, which eventually contributes to vascular degeneration and vascular remodeling via abnormal self-proliferation and promotes the vascular stiffness by the excessive deposition of collagen and decrease of elastin.^15^ Therefore, we hypothesize that endothelial PKR-mediated inflammatory factors release can induce the phenotype transforming of VSMCs to induce vascular aging.

Taken together, we hypothesize that endothelial PKR induces GSDMD-mediated endothelial cell hyperactivation to release HMGB1 and IL-1β, which lead to the phenotype transforming of VSMCs and subsequent accelerates the process of vascular aging.

## Results

### Activity of PKR is elevated in aging vascular endothelium

**Figure 1:**
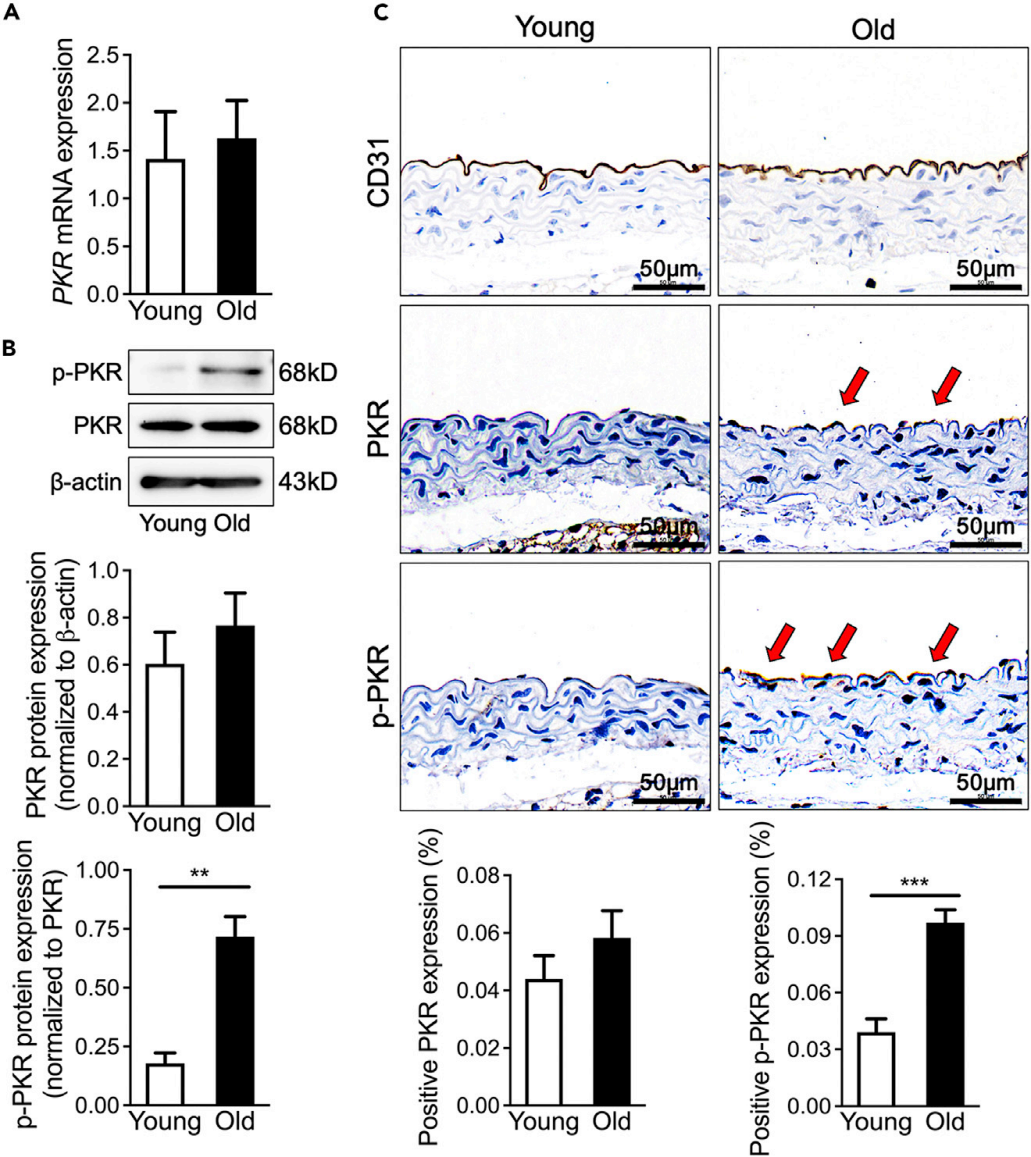
Activity of PKR is elevated in aging vascular endothelium (A) PKR mRNA expression determined by real-time qPCR in young and old WT mice (n = 6). Data are represented as mean ± SEM. (B) Representative immunoblots and densitometric analysis of PKR and *p*-PKR level in young and old WT mice aortas (n = 5). Data are represented as mean ± SEM. (C) Representative photomicrographs and statistical analysis of serial sections of arteries from young and old WT mice (n = 5). Sections were immunohistochemical stained for CD31, PKR and *p*-PKR. Data are represented as mean ± SEM and Scale Bar = 50 μm. ^∗∗^p< 0.01; ^∗∗∗^p< 0.001 (unpaired/two-tailed t-test).

Our previous studies have revealed that PKR activation plays an important role in promoting human umbilical vein endothelial cells (HUVECs) senescence, but the role of PKR in regulating vascular aging is still unknown. To examine whether PKR contributes to vascular aging, qPCR and western blotting were performed in the thoracic aorta from 2-month-old mice (young mice) and 18-month-old mice (aging mice). Inconsistent with our previous studies that PKR expression and activity is elevated in aging HUVECs,^9^ the elevated level of PKR activity also does not accompany an increase of PKR expression in aging thoracic aorta (Figures 1A and 1B). An increased PKR activation is mainly focused on the vascular intima (Figure 1C). These results suggested that the elevated endothelial PKR activity may play important role in vascular aging.

### Knockout of PKR delays vascular aging

**Figure 2:**
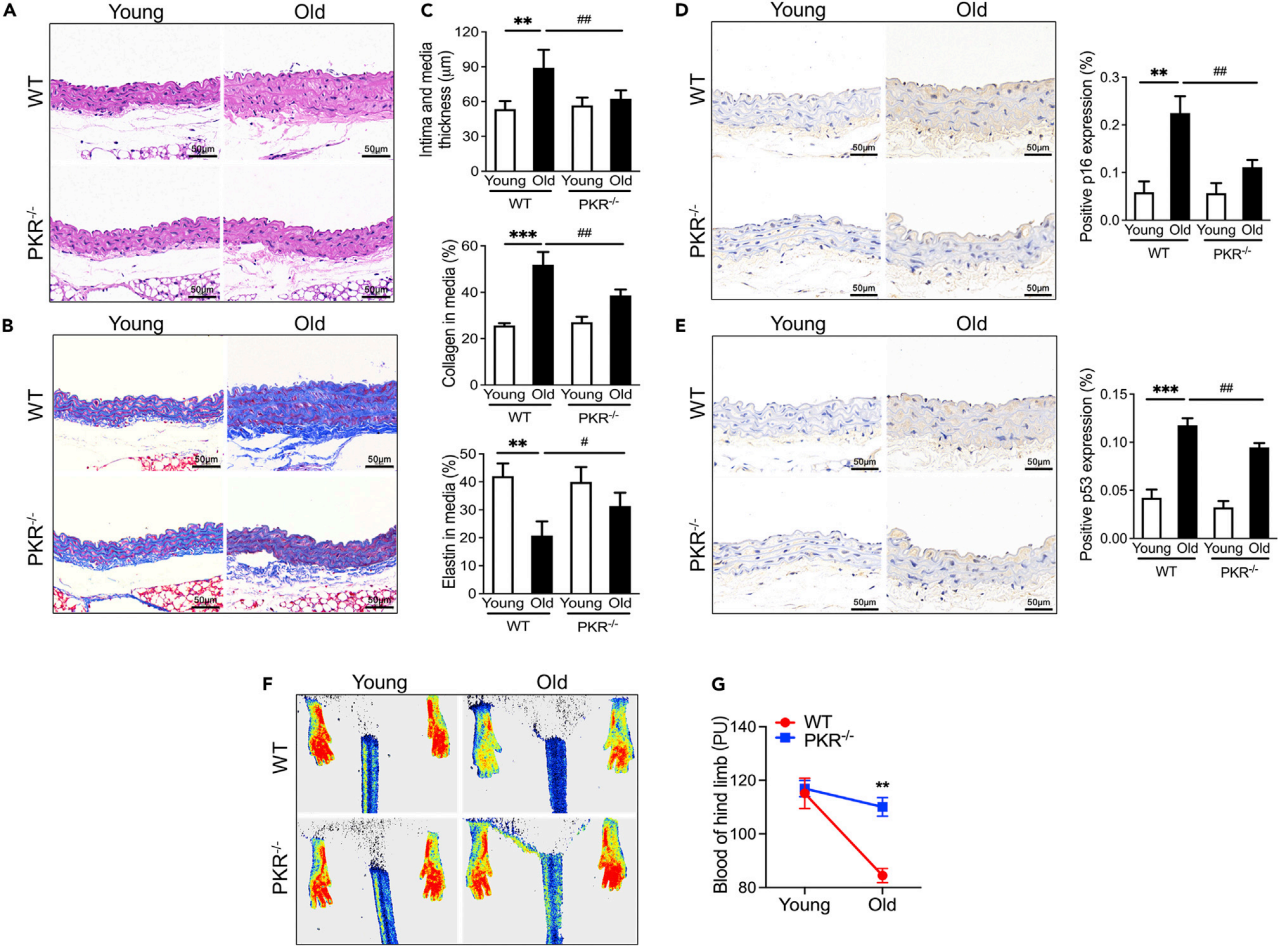
Knockout of PKR delays vascular aging (A) H&E staining of aorta tissues in WT and PKR knockout mice of different age. (B) Masson staining of aorta tissues in WT and PKR knockout mice of different age. (C) Morphometry analysis quantifying intima and media thickness, collagen area and elastin area in the aorta tissues from WT and PKR knockout mice of different age. Data are represented as mean ± SEM. (D) Representative photomicrographs and statistical analysis of serial sections of arteries from WT and PKR knockout mice of different age (n = 5). Sections were immunohistochemical stained for p16 (n = 5). Data are represented as mean ± SEM and Scale Bar = 50 μm. (E) Representative photomicrographs and statistical analysis of serial sections of arteries from WT and PKR knockout mice of different age (n = 5). Sections were immunohistochemical stained for p53 (n = 5). Data are represented as mean ± SEM and Scale Bar = 50 μm. (F) Representative photomicrographs of blood flow density imaged by laser Doppler in the lower limb of WT and PKR knockout mice of different age. (G) Statistical analysis of blood flow density in the lower limb from WT and PKR knockout mice of different ages. Data are represented as mean ± SEM. ^∗∗^p< 0.01; ^∗∗∗^p< 0.001; ^#^p< 0.05; ^##^p< 0.01 (two-way ANOVA test).

To clarify the role of PKR in the vascular aging process, we compared the thoracic aorta morphological difference between 18-month-old PKR knockout mice with the same-month-old wildtype (WT) mice. As revealed by hematoxylin-eosin (H&E) and Masson staining, the thoracic aorta tissue from aging WT mice exhibited obvious vascular aging characteristics, as shown by increase of intima and media thickness (IMT) with increased collagen and decreased elastin content in media (Figures 2A–2C). However, aging PKR knockout mice appeared the alleviative thickening, collagen deposition and elastin decreasing in thoracic aorta tissue when compared to aging WT controls (Figures 2A–2C). At the same time, pro-senescence protein p53 and p16 as senescence-associated markers were also detected by immunohistochemical staining in vascular tissues. We found that pro-senescence protein p53 and p16 both strikingly increased in aging WT mice rather than aging PKR knockout mice (Figures 2D and 2E). In addition, vascular aging is always accompanied with the decline of capillary density and blood flow.^16^ Hence, blood flow intensity was evaluated in the hind limbs of mice by laser Doppler. Compared with young WT mice, the significantly decreased capillary density and blood flow in the hind limbs was shown inold WT mice (Figures 2F and 2G). However, slightly decreased capillary density and blood flow of old PKR knockout mice when compared to young PKR knockout mice (Figures 2F and 2G). These results suggest that PKR knockout can prevent vascular aging process and protect aging-induced vascular dysfunction.

### Knockout of PKR inhibits the transforming phenotype of VSMC in aging vascular

**Figure 3:**
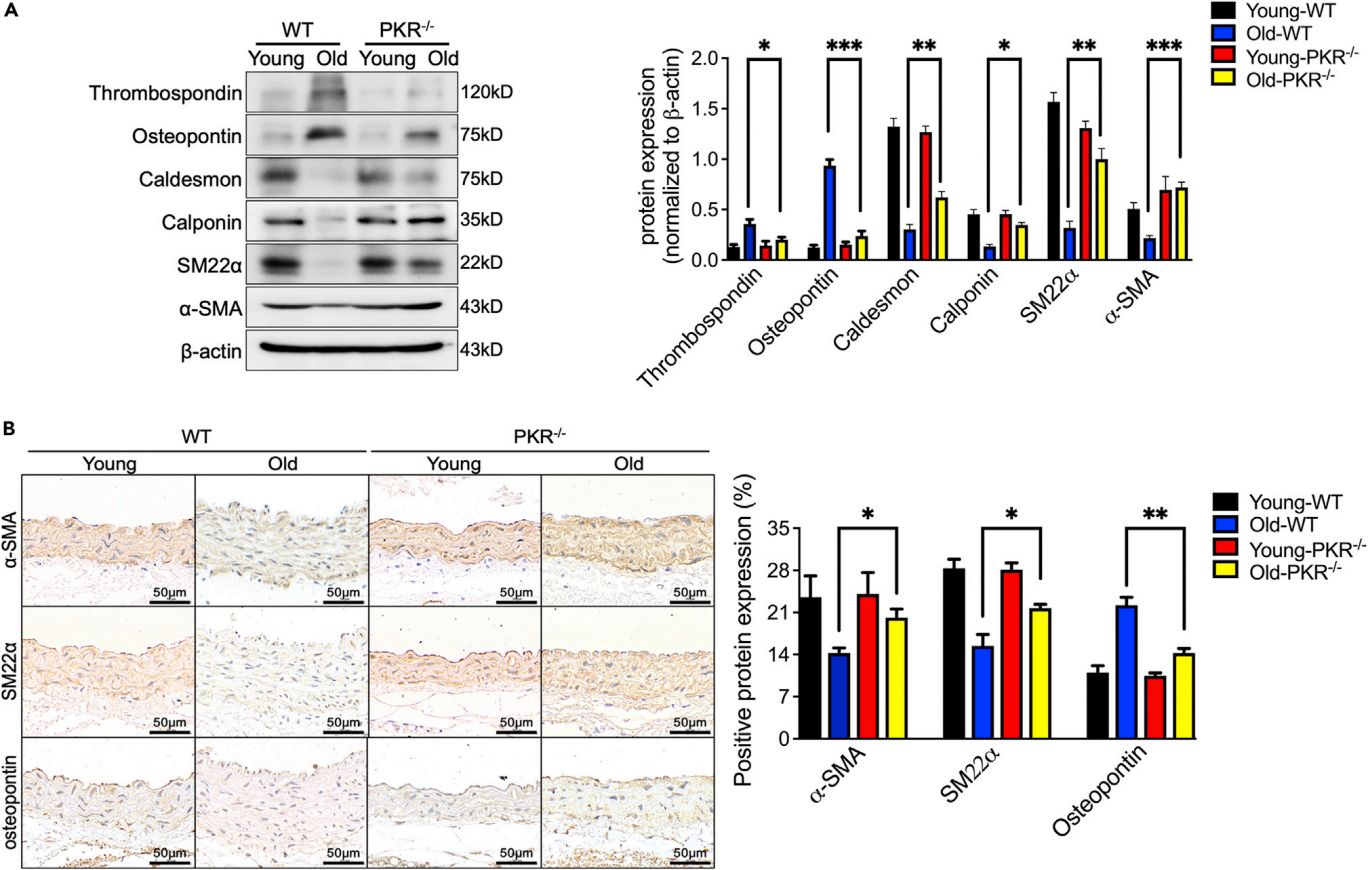
Knockout of PKR inhibits the transforming phenotype of VSMC in aging vessel (A) Representative immunoblots and densitometric analysis of arterial α-SMA, SM22α, caldesmon, calponin, thrombospondin and osteopontin level in WT and PKR knockout mice of different age (n = 4). (B) Representative photomicrographs and statistical analysis of serial sections of arteries from WT and PKR knockout mice of different age (n = 5). Sections were immunohistochemical stained for the α-SMA, SM22α and osteopontin (n = 5). Scale Bar = 50 μm. ^∗^p< 0.05; ^∗∗^p< 0.01; ^∗∗∗^p< 0.001 (two-way ANOVA test). Data are represented as mean ± SEM.

As a prominent phenotype of aging aortas, the contractile phenotype of VSMCs was gradually replaced by the synthetic phenotype of VSMCs and subsequently caused the aortic wall thickening and stiffening in aging mammals.^17^ To clarify the underlying mechanisms of whether PKR induced vascular aging by promoting the transforming phenotype of arterial VSMCs, western blotting showed markedly decreased expression of α-smooth muscle actin (α-SMA), smooth muscle 22 alpha (SM22α), caldesmon and calponin (contractile phenotype markers) with increased level of thrombospondin and osteopontin (synthetic phenotype markers) in aorta of aging WT mice when compared with young WT mice (Figure 3A). However, the aortas from aging PKR knockout mice did not show an obvious switch of synthetic phenotype to contractile phenotype in VSMCs. We also confirmed this phenomenon using immunohistochemical staining to detect the level of α-SMA, SM22α (contractile phenotype markers) and osteopontin (synthetic phenotype markers) (Figure 3B). These results suggested that PKR promotes the transforming phenotype of VSMCs to induce vascular aging.

### Interfering PKR reduces the release of IL-1β and HMGB1

**Figure 4:**
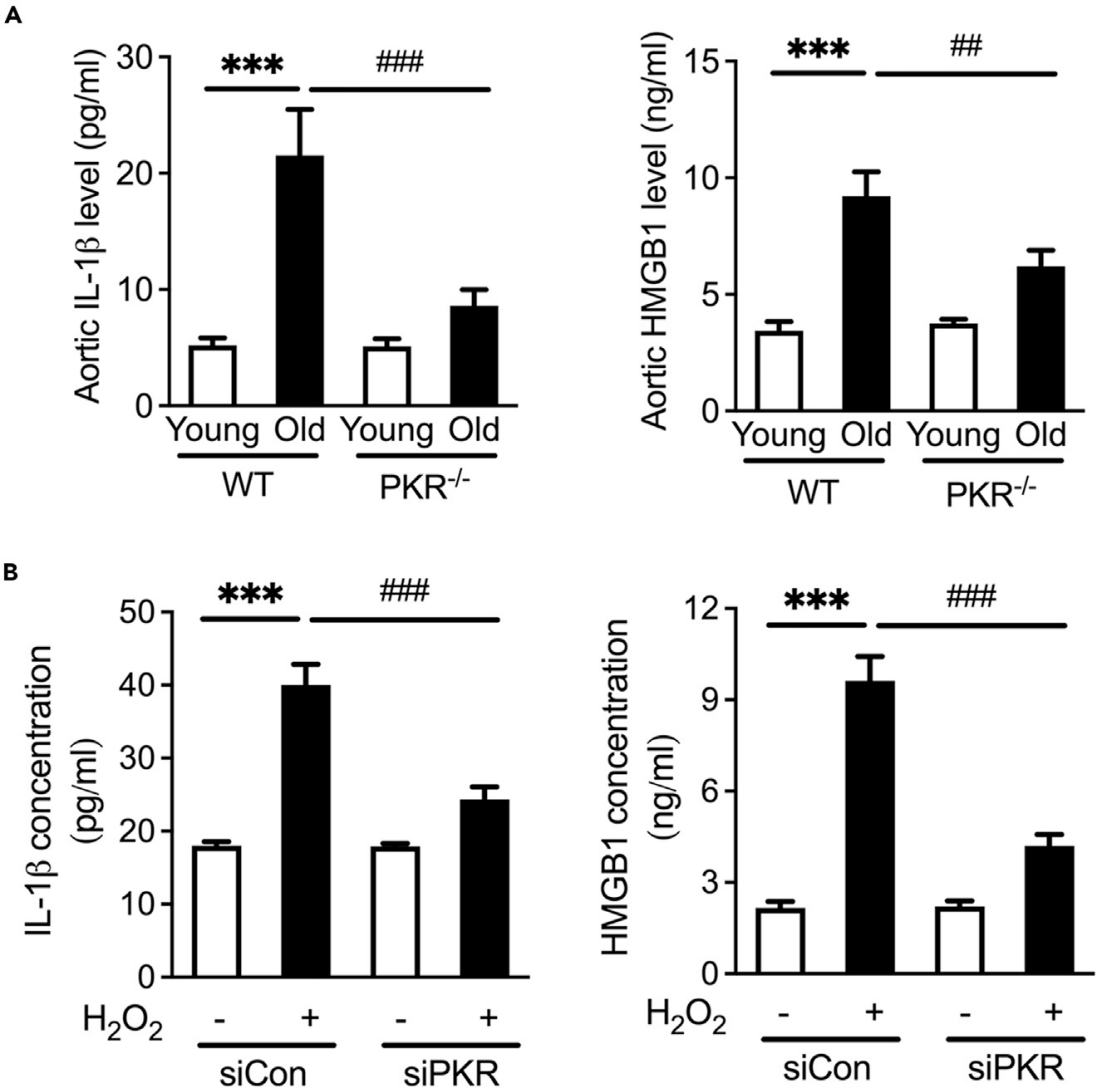
Interfering PKR reduces the release of IL-1β and HMGB1 (A) Aortic IL-1β and HMGB1 level in WT and PKR knockout mice of different age (n = 5). (B) IL-1β and HMGB1 secretion in siCon and PKR knockdown (siPKR) HUVECs stimulated with H_2_O_2_ (n = 3). ^∗∗∗^p< 0.001; ^##^p< 0.01; ^###^p< 0.001 (two-way ANOVA test). Data are represented as mean ± SEM.

We next investigated the mechanisms about how endothelial PKR promotes VSMCs phenotype transformation. Because inflammatory cytokines released from endothelial cells are major drivers of vascular aging,^18^ we measured the cytokine levels in the plasma using ELISA. In previous studies, PKR has been reported to mediate the release of inflammatory cytokines including IL-1β and HMGB1, both of which have been verified to contribute to the process of aging.^19^^,^^20^ We found that the IL-1β and HMGB1 concentration increased in the aorta of aging WT mice (Figure 4A). Notably, their levels in aging PKR knockout mice aorta are not obviously altered. However, we still need to further verify whether PKR from senescence endothelial cell induced the increased IL-1β and HMGB1 release. We suppressed PKR expression using small interfering RNA (siRNA) and demonstrated that PKR silence diminished pro-senescence stimulus H_2_O_2_ mediated IL-1β and HMGB1 release in HUVECs (Figure 4B).

### Inhibiting PKR decreases GSDMD-mediated endothelial cell hyperactivation to prevent inflammatory factors release

**Figure 5:**
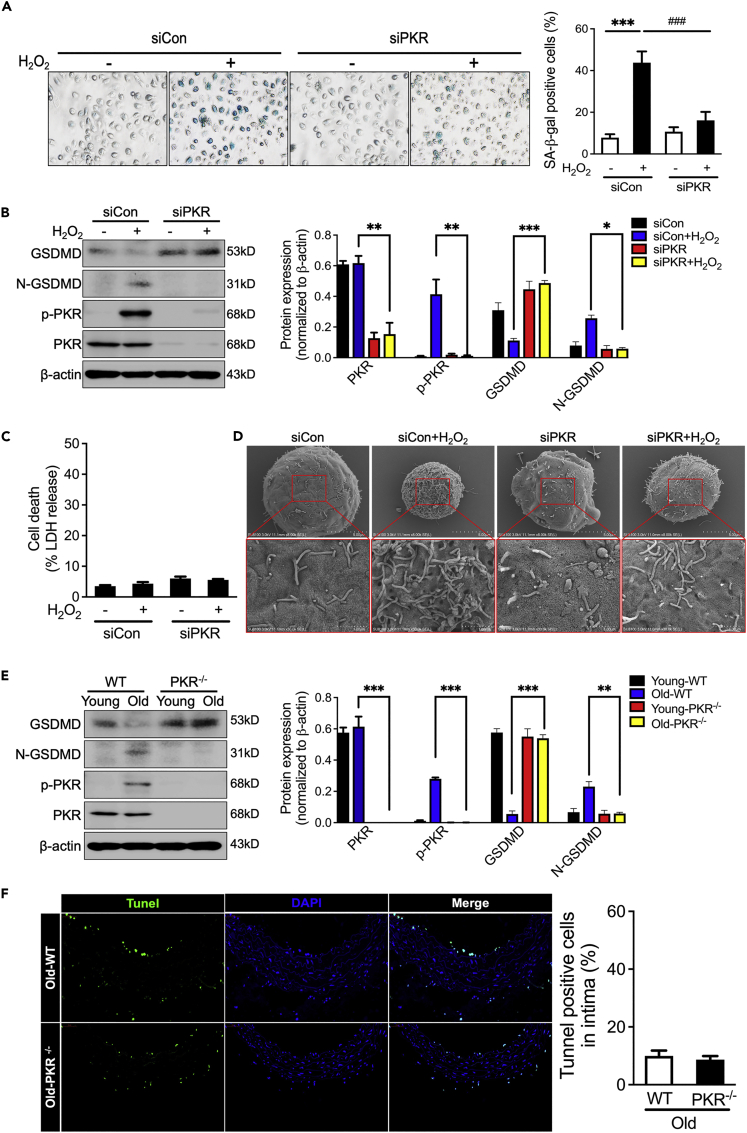
Inhibiting PKR decreases GSDMD-mediated endothelial cell hyperactivation (A) Representative micrographs and positive rate analysis of senescence-associated β-galactosidase (SA-β-gal) staining in siCon and PKR knockdown (siPKR) HUVECs stimulated with H_2_O_2_ (n = 3). (B) Representative immunoblots and densitometric analysis of GSDMD, N-GSDMD, PKR and *p*-PKR level in siCon and PKR knockdown (siPKR) HUVECs stimulated with H_2_O_2_ (n = 3). (C) LDH assay in the supernatants of siCon and PKR knockdown (siPKR) HUVECs stimulated with H_2_O_2_ (n = 3). (D) Representative scanning electronic micrographs of siCon and PKR knockdown (siPKR) HUVECs stimulated with H_2_O_2_. (E) Representative immunoblots and densitometric analysis of arterial β-actin, GSDMD, N-GSDMD, PKR and *p*-PKR level in young and old WT or PKR knockout mice (n = 4). (F) Tunnel staining of aorta in aging WT or PKR knockout mice (n = 4). Scale Bar = 50 μm. ^∗^p< 0.05; ^∗∗^p< 0.01; ^∗∗∗^p< 0.001; ^###^p< 0.001 (two-way ANOVA test). Data are represented as mean ± SEM.

Previous studies have reported that PKR promotes IL-1β and HMGB1 release in pyroptotic cell death. Hence, we detected whether PKR promotes pyroptosis in aging endothelial cells. Endothelial cells were stimulated with H_2_O_2_ to induce senescence phenotype and PKR activation (Figures 5A and 5B). However, unlike pyroptotic cell with GSDMD N-terminal (N-GSDMD) fragment formation and significant LDH release, endothelial cells with H_2_O_2_ stimulation induce GSDMD N-terminal (N-GSDMD) fragment formation without LDH release (Figures 5B and 5C). The HUVECs morphology was observed by a scanning electron microscope. Compared with untreated cells, H_2_O_2_ stimulation induced a lot more pore formation in cell membrane (Figure 5D). In addition, similar results were demonstrated in aging vascular tissues. Although increased N-GSDMD was found in aging vascular tissues (Figure 5E), but tunnel staining did not demonstrate increased cell death in aging PKR knockout mice of vascular endothelium (Figure 5F). Despite this, knockdown of PKR effectively inhibited GSDMD N-terminal (N-GSDMD) fragment formation and pore-formation in HUVECs (Figures 5D and 5E). Taken together with the above results, we suggested that senescent endothelial cells are accompanied by cell hyperactivation rather than cell pyroptosis. Lacking PKR can block the development of cell hyperactivation in senescent endothelial cells.

**Figure 6:**
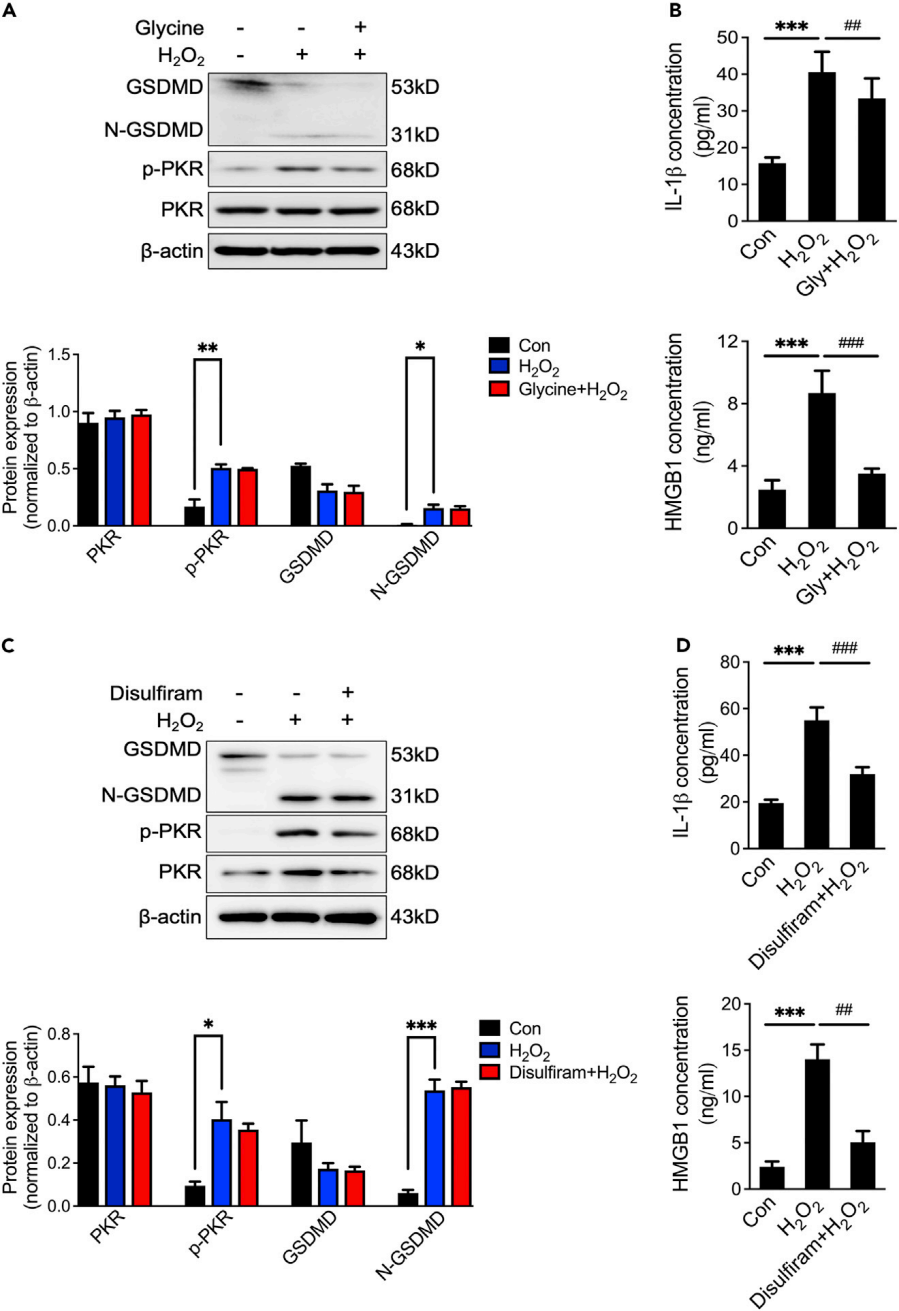
Inhibiting endothelial cell hyperactivation prevents the release of IL-1β and HMGB1 (A) Representative immunoblots and densitometric analysis of GSDMD, N-GSDMD, PKR and *p*-PKR levels in HUVECs with glycine priming and then stimulated with H_2_O_2_ (n = 3). (B) IL-1β and HMGB1 secretion in HUVECs with glycine priming and then stimulated with H_2_O_2_ (n = 3). (C) Representative immunoblots and densitometric analysis of GSDMD, N-GSDMD, PKR and *p*-PKR level in HUVECs with disulfiram priming and then stimulated with H_2_O_2_ (n = 3). (D) IL-1β and HMGB1 secretion in HUVECs with disulfiram priming and then stimulated with H_2_O_2_ (n = 3). ^∗∗∗^p< 0.001; ^##^p< 0.01; ^###^p< 0.001 (one-way ANOVA test). Data are represented as mean ± SEM.

To further confirm that GSDMD-mediated endothelial cell hyperactivation promotes the release of inflammatory factors, osmoprotectant glycine, known as an inhibitor of hyperactivation, was used to prevent membrane rupture and pyroptosis-induced cell lysis (Fink and Cookson, 2006). Compared with H_2_O_2_-treated group, glycine treatment did not decrease activation of GSDMD (Figure 6A), but glycine greatly reduced the release of IL-1β and HMGB1 (Figure 6B). In addition, an FDA-approved GSDMD pore formation inhibitor disulfiram was used to prevent H_2_O_2_-induced membrane rupture.^21^ Similar to glycine, disulfiram failed to inhibit H_2_O_2_-induced cleavage of GSDMD but hindered the release of IL-1β and HMGB1 (Figures 6C and 6D). These results indicate that PKR promotes GSDMD-mediated endothelial cell hyperactivation and subsequent inflammatory factors release.

### Silencing endothelial PKR inhibits the phenotypic transformation of vascular smooth muscle

**Figure 7:**
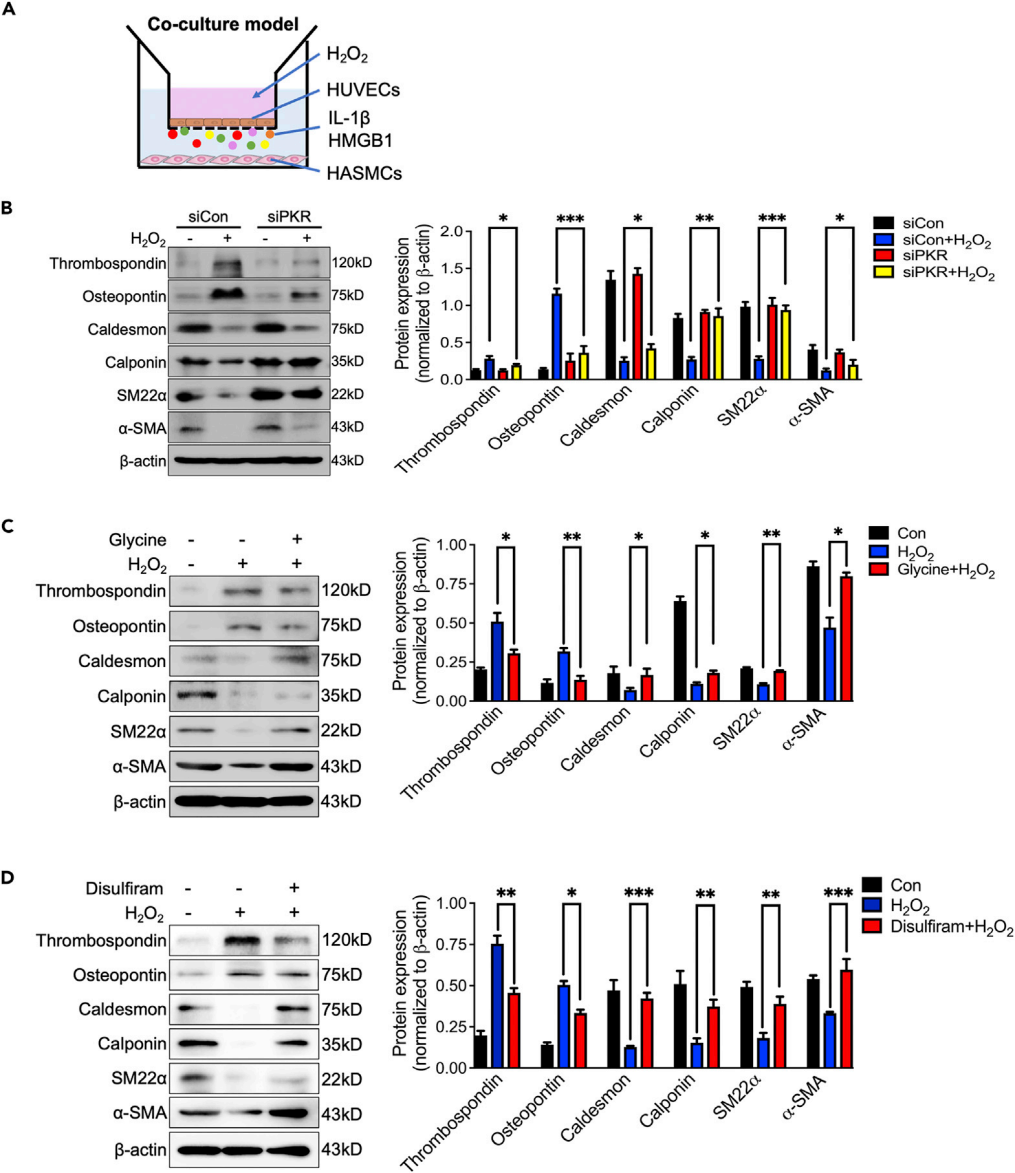
Silencing endothelial PKR inhibits the phenotypic transformation of vascular smooth muscle (A) The diagram of co-culture system. (B) Representative immunoblots and densitometric analysis of α-SMA, SM22α, caldesmon, calponin, thrombospondin and osteopontin level in HASMCs that co-cultured with indicated HUVECs (n = 3). (C) Representative immunoblots and densitometric analysis of α-SMA, SM22α, caldesmon, calponin, thrombospondin and osteopontin level in HASMCs that co-cultured with indicated HUVECs (n = 3). (D) Representative immunoblots and densitometric analysis of α-SMA, SM22α, caldesmon, calponin, thrombospondin and osteopontin level in HASMCs that co-cultured with indicated HUVECs (n = 3). ^∗^p< 0.05; ^∗∗^p< 0.01; ^∗∗∗^p< 0.001 (two-way ANOVA test). Data are represented as mean ± SEM.

We further co-cultured HUVECs with HASMCs to investigate whether activated PKR-induced endothelial hyperactivation can trigger VSMCs phenotypic transformation (Figure 7A). The phenotypic transformation of co-cultured HASMCs was detected after silencing endothelial PKR expression by siRNA and then stimulated with H_2_O_2_. Results showed that normal endothelial cells cannot induce the phenotypic transforming of HASMCs, but H_2_O_2_-induced senescent endothelial cell promotes HASMCs phenotypic transformation by decreasing levels of α-SMA, SM22α, calponin and caldesmon with increasing level of thrombospondin and osteopontin (Figure 7B). Knockdown of endothelial PKR significantly reversed H_2_O_2_-induced HASMCs phenotypic transformation (Figure 7B). Moreover, performing glycine or disulfiram in H_2_O_2_-stimulated HUVECs also can inhibit the phenotypic transforming of co-cultured HASMCs (Figures 7C and 7D).

### Silencing endothelial PKR inhibits palmitic acid-induced endothelial cell hyperactivation and phenotypic transformation of vascular smooth muscle

**Figure 8:**
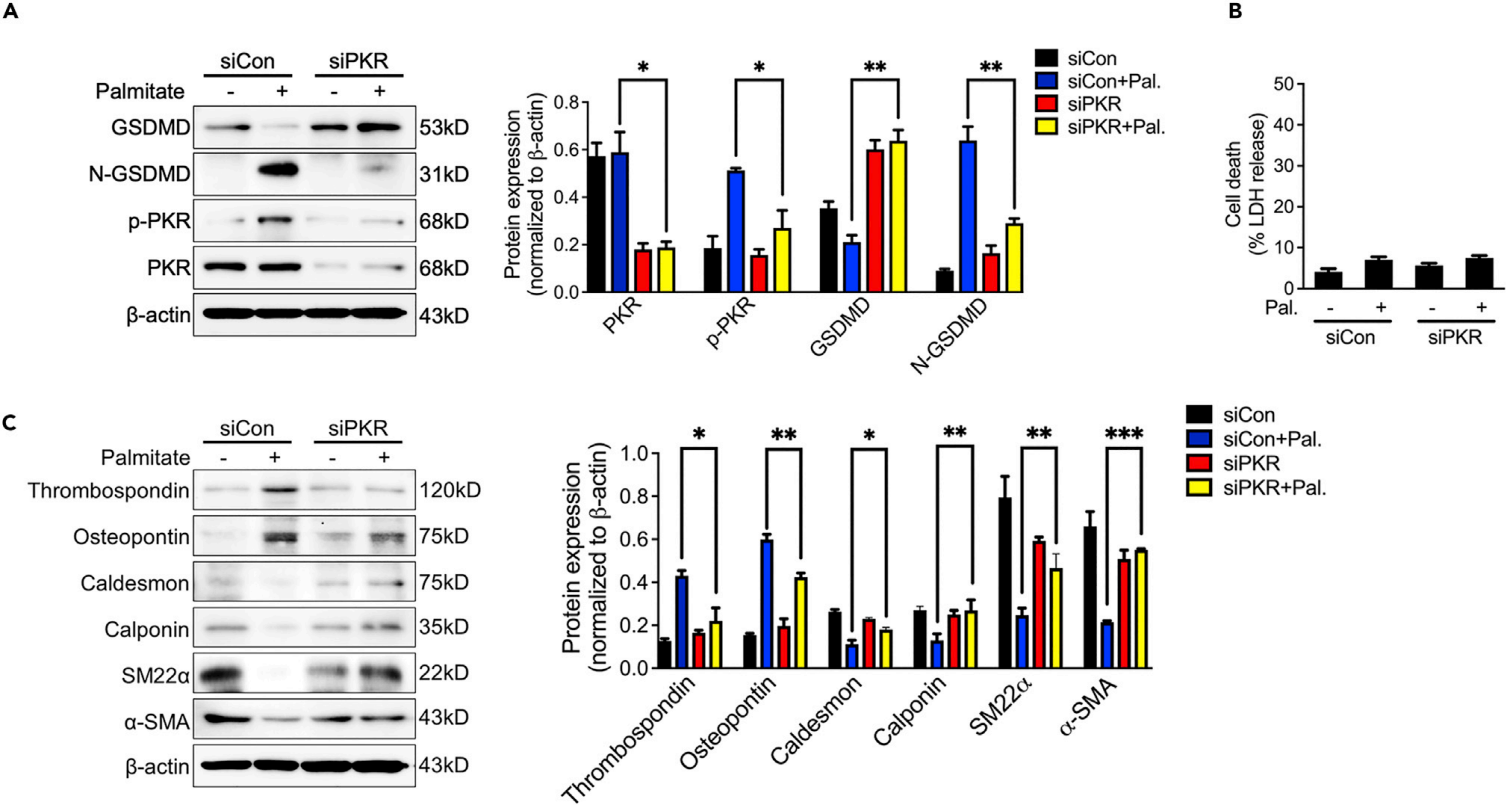
Silencing endothelial PKR inhibits palmitic acid-induced endothelial cell hyperactivation and phenotypic transformation of vascular smooth muscle (A) Representative immunoblots and densitometric analysis of GSDMD, N-GSDMD, PKR and *p*-PKR level in siCon and PKR knockdown (siPKR) HUVECs stimulated with palmitic acid (n = 3). (B) LDH assay in the supernatants of siCon and PKR knockdown (siPKR) HUVECs stimulated with palmitic acid (n = 3). (C) Representative immunoblots and densitometric analysis of α-SMA, SM22α, caldesmon, calponin, thrombospondin and osteopontin level in HASMCs that co-cultured with indicated HUVECs (n = 3). ^∗^p< 0.05; ^∗∗^p< 0.01; ^∗∗∗^p< 0.001 (two-way ANOVA test). Data are represented as mean ± SEM.

Elevated palmitate acid (PA) level is an independent risk factor of cardiovascular diseases by promoting vascular endothelial cell senescence.^9^ However, the role of PA on inducing endothelial cell hyperactivation and phenotypic transformation of vascular smooth muscle is still unknown. Similar to H_2_O_2_ stimulation, PA also triggered endothelial cell hyperactivation and phenotypic transformation of co-cultured vascular smooth muscle by shearing GSDMD to N-GSDMD without LDH release and decreasing levels of α-SMA, SM22α, calponin and caldesmon with increasing level of thrombospondin and osteopontin (Figures 8A–8C). Knockdown of PKR by siRNA also inhibited PA-induced endothelial cell hyperactivation and phenotypic transformation of co-cultured vascular smooth muscle (Figures 8A–8C). Combined with above results, we suggested that silencing endothelial PKR inhibits the phenotypic transformation of vascular smooth muscle via blocking GSDMD-mediated endothelial cell hyperactivation, which may eventually repress the process of vascular aging.

## Discussion

Vascular aging is the major risk factor of aging-associated CVDs and atherosclerosis. Understanding the molecule mechanism of vascular aging may provide potential therapeutic strategies and targets for the treatment of aging-associated CVDs. In the present study, we made four important findings. (1) We demonstrated that PKR activation was increased in aging vascular intima. (2) Deficiency of PKR alleviated aging-induced vascular aging characterized as vascular remodeling, collagen deposition, senescent related signaling alteration, and decreased of blood flow. (3) The protection was verified by inhibiting GSDMD-mediated endothelial hyperactivation and inflammatory factors (IL-1β and HMGB1) release. (4) Activated PKR-induced endothelial cell hyperactivation promotes phenotypic transformation of smooth muscle cells and eventually contributes to vascular aging. Our research revealed a vital role of PKR in the progress of vascular aging, and the mechanism is GSDMD-mediated endothelial cell hyperactivation and subsequent inflammatory factors release which lead to the phenotype transforming of HASMCs, and this is the first to elucidate the crosstalk between endothelial cell injury and SMCs abnormal function. These findings provide a new insight into vascular aging and shed light on the intervention of aging-associated cardiovascular disease.

Previous studies proved that PKR is associated with neuroaging-related diseases, including Alzheimer’s disease and neurodegenerative diseases. Our study further found that PKR also can promote vascular aging. Based on the results that the activity of PKR is especially increased in vascular endothelium and that global knockout of PKR sufficiently delays the vascular aging, we speculated that endothelial PKR is a key target of vascular aging. Aging endothelium is easily attacked by cytokines, inflammatory, metabolic products, and shearing force, all of which are also activators of PKR phosphorylation. PKR, as a crucial stress-responsive kinase, can be activated by virus, bacteria, cytokines, inflammatory factors, and metabolic products, even including oxidative stress and metabolic stress.^22^ Our results may suggest that endothelial PKR can be activated by a variety of stress factors, which is the initial link to induce vascular aging.

However, we cannot totally exclude that PKR in blood cells also contributes to the progression of vascular aging. In previous studies, innate immune cells also associated the development of vascular aging. Abnormal monocyte-macrophages, neutrophils, T cells and dendritic cells are prominent in aging vascular remodeling.^23^ Because PKR has long been verified to promote inflammatory factors release in immune cells,^20^ whether the pro-inflammatory role of PKR in immune cells also promotes the development of vascular aging still requires further study.

Our results supported that PKR promotes GSDMD activation in aging vessels. However, unlike previous studies that reported that activated GSDMD mediates the development of pyroptosis, we found that PKR induces GSDMD-mediated cell hyperactivation in aging endothelial cells. In pyroptotic cells, GSDMD triggers plasma membrane rupture (PMR) and cell death, which is accompanied by the leakage of IL-1, HMGB1, other cytokines, and LDH. However, GSDMD mediated the secretion of IL-1 and other cytokines without LDH in living hyperactivated cells. We speculated that the reason why PKR induces hyperactivation but not pyroptosis in endothelial cells is because PKR cannot further spur GSDMD-dependent nerve injury–induced protein 1 (NINJ1) activation.^24^ NINJ1, as a membrane protein, can be activated by caspase-11/GSDMD signaling and then lead to PMR, which causes pyroptotic cell death.^25^ However, we speculated that overactivated PKR fails to mimic caspase11/GSDMD signaling mediated NINJ1 activation in aging endothelial cells, suggesting that endothelial inflammatory factors release rather than cell death is more important in promoting vascular aging.

In addition, previous studies reported that performing membrane rupture inhibitor glycine to pharmacologically inhibit GSDMD mediated pore formation can artificially switch LPS plus Nigericin mediated macrophage pyroptosis to cell hyperactivation.^13^ Subsequent studies demonstrated that hyperactivated macrophages can secrete fibrin to promote disseminated intravascular coagulation (DIC) in endotoxemic mice.^26^ Our results confirm that in endothelial cells, PKR/GSDMD pathway can specifically induce the hyperactivation of endothelial cells and induce vascular aging by releasing various inflammations and cytokines. The results confirmed that cell hyperactivation also played an important role in endothelial cells and provided evidence for explaining the process of endothelial vascular aging.

In summary, the study confirmed that PKR has an essential role in the process of vascular aging. Given the global knockout of PKR effectively delaying vascular aging, targeting PKR may be of therapeutic benefit to vascular aging in clinical patients, but whether pharmacological inhibition of PKR delays vascular aging still needs to be revealed in further studies *in vivo* and clinically.

### Limitation of the study

We demonstrated that endothelial PKR plays an important role in promoting vascular aging by PKR-global knockout mice and PKR siRNA in cells. However, this result should be further confirmed by specific PKR endothelial knockout mice and PKR knockout endothelial cells. Because PKR from innate immune cells also promotes the release of HMGB1 and IL-1β which may mediate phenotypic transformation of vascular smooth muscle, we admitted that using HAoECs but not HUVECs is more suited to explain the mechanism of this study. In addition, the detection of PKR activity in clinical vascular samples is lacking, and the pro-vascular aging role of PKR in clinical patients still requires further study.

## STAR★Methods

### Key resources table

**Table:** 

REAGENT or RESOURCE	SOURCE	IDENTIFIER
**Antibodies**		

Anti-PKR antibody	Abcam	Cat#: ab32052; RRID: AB_2293421
Anti-p-PKR antibody	Abcam	Cat#: ab32036; RRID: AB_777310
Anti-GSDMD antibody	Abcam	Cat#: ab210070; RRID: AB_2893325
Anti-N-GSDMD antibody (EPR20829-408)	Abcam	Cat#: ab215203; RRID: AB_2916166
Anti-IL-1β antibody	R&D system	Cat#: AF-401-NA; RRID: AB_416684
Anti-α-SMA antibody	Proteintech	Cat#: 14395-1-AP; RRID: AB_2223009
Anti-CD31 antibody	Abcam	Cat#: ab182981; RRID: AB_2920881
Anti-SM22α antibody	Proteintech	Cat#:10493-1-AP; RRID: AB_2199363
Anti-Calponin antibody	Proteintech	Cat#:13938-1-AP; RRID: AB_2082010
Anti-Caldesmon antibody	Proteintech	Cat#: 20887-1-AP; RRID: AB_10792408
Anti-Osteopontin antibody	Proteintech	Cat#: 22952-1-AP; RRID: AB_2783651
Anti-Thrombospondin antibody	Proteintech	Cat#: 18304-1-AP; RRID: AB_2201959
Anti-P53 antibody	Proteintech	Cat#: 10442-1-AP; RRID: AB_2206609
Anti-P16^INK4a^ antibody	Proteintech	Cat#: 10883-1-AP; RRID: AB_2078303
β-actin antibody	Cell Signaling Technologies	Cat#: 3700S; RRID: AB_2242334

**Chemicals, peptides, and recombinant proteins**		

H_2_O_2_	Sinopharm Chemical Reagent	Cat#:10011208
palmitate acid	Sigma-Aldrich	Cat#: P9767
disulfiram	Selleck	Cat#: S1680
glycine	Genview	Cat#: FG149

**Critical commercial assays**		

Mouse IL-1β ELISA Kit	R&D Systems	Cat#: MLB00C
Human IL-1β ELISA Kit	Abcam	Cat#: ab214025
HMGB1 ELISA Kit	Shino-Test Corp.	Cat#:ST51011
β-gal staining solution	Beyotime BioTechnology	Cat#:C0602
RIPA lysis buffer	Beyotime BioTechnology	Cat#: P0013B
Protease inhibitor cocktail	Roche Diagnostics	Cat#: 5892791001
BCA protein assay kit	Pierce	Cat#: 23225
RNA easy Isolation Reagent	Vazyme	Cat#: R701-01
Lipofectamine 3000 transfection reagent	Invitrogen	Cat#: L3000-015

**Deposited data**		

PKR siRNA	RiboBio	Decribed in current manuscript
Negative control siRNA	RiboBio	Decribed in current manuscript
Human PKR primer	Sangon bio	Decribed in current manuscript
Human BETA-ACTIN primer	Sangon bio	Decribed in current manuscript

**Experimental models: Cell lines**		

HUVECs	ATCC	Cat#: CRL-1730, RRID: CVCL_2959
HASMCs	ATCC	Cat#: CRL-1999, RRID: CVCL_4009

**Experimental models: Organisms/strains**		

C57BL/6 mice	Jackson Laboratory	000664
PKR^−/−^ mice	John C. Bell Lab	Abraham et al.^27^

**Software and algorithms**		

Moor Laser Doppler Imager	Moor Instruments	https://www.moor.co.uk/products/imaging/high-resolution-laser-doppler-imaging/
Scanning electron microscopy	Hitachi	https://cmrf.research.uiowa.edu/hitachi-s-3400n
Image-ProPlus v.6.0	MediaCybernetics	http://www.mediacy.com/imageproplus
ImageJ	NIH	https://imagej.nih.gov/ij/index.html
GraphPad Prism v.5	GraphPad Software	https://www.graphpad.com/scientific-software/prism/
LEGENDplex Data Analysis Software	BioLegend	https://www.biolegend.com/legendplex/
SPSS v.26	IBM	https://www.ibm.com/cn-zh/spss

### Resource availability

#### Lead contact

Further information and requests for resources and reagents should be directed to and will be fulfilled by the lead contact, Yapei Li (lypei123@163.com).

#### Materials availability

This study did not generate new unique reagents.

### Experimental model and subject details

#### Animals

Animal care and experiments were performed with approval from the animal ethics committees of Central South University, and all the experimental procedures were conducted in accordance with the NIH Guide for the Care and Use of Laboratory Animals. PKR knockout mice were kindly provided by Dr. Jianfeng Wu (Xiamen University, Xiamen, China). PKR knockout mice are genotyped with PCR primers (240-base-pair (bp) wild-type DNA band: forward, 5′-GGAACTTTGGAGCAATGGA-3′, reverse, 5′-TGCCAATCACAAATCTAAAAC-3′ and 460-bp mutant DNA band: forward, 5′-TGTTCTGTGGCTATCAGGG-3′, reverse, 5′-TGACGAGTTCTTCTGAGGG-3′) in 2% agarose gel in TAE buffer. The male PKR knockout mice were used in this study. C57/B6L male mice were used as wild-type mice. Mice were anesthetized with pentobarbital sodium (65 mg/kg i.p.) to measure the blood flow of the limbs and obtain aorta tissues. Other mice not subjected to tissue harvesting were euthanized by cervical dislocation after inhalation of carbon dioxide.

#### Cell culture and treatment

Human umbilical vein endothelial cell (HUVECs) and Human aortic smooth muscle cell (HASMCs) were purchased from AmericanType Culture Collection. HUVECs were cultured in EC growth medium bullet kit (EGM-2, Lonza). HASMCs were grown in smooth muscle cell growth medium-2 (SmGM-2, Lonza). Both cells were not used beyond passage 15. H_2_O_2_ (Sinopharm Chemical Reagent Co., Ltd) was used to induce endothelial cell senescence at a concentration of 100μM for 8 h. Palmitate acid (Sigma-Aldrich) was used to induce cell senescence at a concentration of 200μM for 16 h. To inhibit the role of H_2_O_2_ or palmitate acid, HUVECs were pretreated with 5 mM glycine (Sigma-Aldrich) for 4 h or 50uM disulfiram (Selleck) for 1h before stimulation with H_2_O_2_ or palmitate acid, and cell lysates and precipitated supernatants were then subjected to the subsequent experiments. LDH assays were performed using LDH-Glo™ Cytotoxicity Assay (Promega) according to the manufacturer’s instructions.

### Method details

#### Histopathological and immunohistochemistry analysis

Sections were fixed in 4% formalin for 48h and then dehydrated, embedded in paraffin and cut into 4μm sections. Morphological changes of aortic walls were observed in sections stained with Hematoxylin-eosin staining. The collagen deposition and elastin content of aortic walls was evaluated with Masson trichrome staining. The media thickness, relative positive staining area for collagen and elastin were measured by an image analysis system (Image-ProPlus, Version 6.0; Media Cybernetics). Immunohistochemistry of tissue sections were performed with the following primary antibodies: anti-PKR (1:100, Abcam), anti-p-PKR (1:100, Abcam), anti-α-SMA (1:100, Proteintech), CD31 (1:100, Abcam), SM22α (1:100, Proteintech), Calponin (1:100, Proteintech), Caldesmon (1:100, Proteintech), Osteopontin (1:100, Proteintech), Thrombospondin (1:100, Proteintech), p53 (1:100, Proteintech), p16^INK4a^ (1:100, Proteintech).

#### Laser doppler imaging

Blood flow of the limbs were measured using a Moor Laser Doppler Imager (Moor Instruments, Ltd., Axminster) at 37 ± 0.5°C under ketamine/xylazine (100 mg/10 kg) anesthesia. The blood flow of the limbs was measured by imaging the flux (blood × area-1 × time-1) and analyzed with Moor Laser Doppler Imaging system. A total of six mice per time point per genotype were analyzed.

#### siRNA transfection

Human HUVECs were maintained in serum-free medium and transfected with PKR siRNA or scramble siRNA using Lipofectamine 3000 transfection reagent (Invitrogen, USA) according to the manufacturer’s instructions. The PKR siRNA (RiboBio) sequence is 5′-CAAAUUAGCUGUUGAGAUA-3′. Negative control siRNA sequence is 5′-CGUACGCGGAAUACUUCGA-3′. Six hours after transfection, the cells were returned to normal culture medium for 48 h prior to the following treatment.

#### Co-culture experiments

To evaluate the effect of endothelial hyperactivation on smooth muscle cell *in vitro*, a cell co-culture model was established. Six-well plates with a 0.4-μm pore-sized filter were used according to the manufacturer’s instructions. HUVECs (5×10^5^ cells/well) were seeded in the upper chamber and allowed to adhere overnight prior to the coculture experiments. And 5×10^5^ cells/well were seeded in the bottom chamber. Before beginning the co-culture experiments, indicated HUVECs were stimulated with H_2_O_2_ and were co-cultured with HASMCs to observe the change of HASMCs phenotype transformation. After 48 h, the phenotype transformation was calculated with western blotting.

#### SA-β-gal staining

HUVECs senescence was determined by SA-β-gal staining. Briefly, after transfection or incubation with the appropriate treatment, HUVECs were fixed with fixation solution for 15 minat room temperature, and cells were then incubated at 37°C without CO_2_ overnight in β-gal staining solution (Beyotime BioTechnology), according to the manufacturer’s instructions. Fields of view for each sample were imaged using an inverted microscope (Olympus). Cells with SA-β-gal-stained cytoplasm (blue color) were considered positive cells. The proportion of positive cells were determined by counting the blue cells and dividing the total number of observed cells.

#### Scanning electron microscopy

Cells grown on glass coverslips were washed with PBS, and then fixed with 3% glutaraldehyde overnight at 4°C, followed by rinsing with PBS for three times. Samples were dehydrated through a graded series of ethanol (50, 70, 80, 95 and 100%) and dried by Critical Point Dryer CPD 300 (Leica). Dried specimens were sputter coated with gold-palladium by SuperCool Sputter Coater SCD050 (Leica) and imaged with a scanning electron microscope S3400N-II (Hitachi) operating at 10 kV.

#### ELISA

Serum or cellular supernatant levels of HMGB1 and IL-1b were measured with ELISA kits (HMGB1 ELISA kit, Shino-Test Corp., Kanagawa, Japan, mouse IL-1b ELISA kit, R&D Systems, USA and Human IL-1β ELISA Kit, Abcam, USA). The procedure was performed following the manufacturer’s instructions.

#### Western blotting

Frozen tissues or cells were lysed with RIPA lysis buffer containing PMSF, protease and phosphatase inhibitors (Roche Diagnostics). Protein concentration was quantified using a BCA protein assay kit (Pierce). Samples containing 30 μg of protein were separated by SDS-PAGE gels and transferred to PVDF membranes. The membranes were blocked with 5% fat-free milk before incubation with primary antibodies specifically recognizing PKR (ab32052, 1:1000, Abcam), p-PKR (ab32036, 1:1000, Abcam), human GSDMD (ab210070, 1:1000, Abcam), rabbit anti-human N-GSDMD antibody (EPR20829-408) (ab215203, 1:1000, Abcam) and IL-1β (MAB201, 1:1000, R&D system), α-SMA (1:1000, Proteintech), SM22α (1:1000, Proteintech), calponin (1:1000, Proteintech), caldesmon (1:1000, Proteintech), thrombospondin (1:1000, Proteintech) and osteopontin (1:1000, Proteintech). Immunoreactivity was detected by an enhanced chemiluminescence system, and bands were scanned with a Bio-Rad Imager. Individual band intensity was determined with ImageJ software.

#### Reverse transcription PCR (RT-PCR)

Total RNA was extracted using the RNA easy kit (Vazyme) according to the manufacturer’s instructions. The SuperScript^TM^ OneStep RT-PCR system (Invitrogen) was used to measure the mRNA levels of PKR. The primer sequences used for real-time quantitative PCR analysis were as follows: human PKR (forward: 5′-CCTGTCCTCTGGTTCTTTTGCT-3′ and reverse 5′-GATGATTCAGAAGCGAGTGTGC-3′) and human BETA-ACTIN (forward: 5′-CATGTACGTTGCTATCCAGGC-3′ and reverse: 5′-CTCCTTAATGTCACGCACGAT-3′).

### Quantification and statistical analysis

Values were expressed as mean ± SEM.Data were analyzed by student’s T-test, one-way analysis of variance (ANOVA) and two-way ANOVA. p ≤ 0.05 was statistically significant.

## Data Availability

This paper does not report original code. The data reported in this paper will be shared by the lead contact upon request. The additional information required to reanalyze the data reported in this paper is available from the lead contact upon request.

## References

[1] Harper S. (2014). Economic and social implications of aging societies. Science.

[2] Ungvari Z., Tarantini S., Donato A.J., Galvan V., Csiszar A. (2018). Mechanisms of vascular aging. Circ. Res..

[3] Donato A.J., Machin D.R., Lesniewski L.A. (2018). Mechanisms of dysfunction in the aging vasculature and role in age-related disease. Circ. Res..

[4] Zhao D., Liu J., Wang M., Zhang X., Zhou M. (2019). Epidemiology of cardiovascular disease in China: current features and implications. Nat. Rev. Cardiol..

[5] Bruno R.M., Nilsson P.M., Engström G., Wadström B.N., Empana J.P., Boutouyrie P., Laurent S. (2020). Early and supernormal vascular aging: clinical characteristics and association with incident cardiovascular events. Hypertension.

[6] Xu H., Li S., Liu Y.S. (2022). Nanoparticles in the diagnosis and treatment of vascular aging and related diseases. Signal Transduct. Target. Ther..

[7] Taylor S.S., Haste N.M., Ghosh G. (2005). PKR and eIF2alpha: integration of kinase dimerization, activation, and substrate docking. Cell.

[8] Liu C.X., Li X., Nan F., Jiang S., Gao X., Guo S.K., Xue W., Cui Y., Dong K., Ding H. (2019). Structure and degradation of circular RNAs regulate PKR activation in innate immunity. Cell.

[9] Li Y., Peng Z., Wang C., Li L., Leng Y., Chen R., Yuan H., Zhou S., Zhang Z., Chen A.F. (2018). Novel role of PKR in palmitate-induced Sirt1 inactivation and endothelial cell senescence. Am. J. Physiol. Heart Circ. Physiol..

[10] Li Y., Li Y., Li L., Yin M., Wang J., Li X. (2021). PKR deficiency alleviates pulmonary hypertension via inducing inflammasome adaptor ASC inactivation. Pulm.Circ..

[11] Bloom S.I., Islam M.T., Lesniewski L.A., Donato A.J. (2023). Mechanisms and consequences of endothelial cell senescence. Nat. Rev. Cardiol..

[12] Broz P., Pelegrín P., Shao F. (2020). The gasdermins, a protein family executing cell death and inflammation. Nat. Rev. Immunol..

[13] Evavold C.L., Ruan J., Tan Y., Xia S., Wu H., Kagan J.C. (2018). The pore-forming protein gasdermin D regulates interleukin-1 secretion from living macrophages. Immunity.

[14] Mistriotis P., Andreadis S.T. (2017). Vascular aging: molecular mechanisms and potential treatments for vascular rejuvenation. Ageing Res. Rev..

[15] Lacolley P., Regnault V., Avolio A.P. (2018). Smooth muscle cell and arterial aging: basic and clinical aspects. Cardiovasc.Res..

[16] Das A., Huang G.X., Bonkowski M.S., Longchamp A., Li C., Schultz M.B., Kim L.J., Osborne B., Joshi S., Lu Y. (2018). Impairment of an endothelial NAD(+)-H2S signaling network is a reversible cause of vascular aging. Cell.

[17] Wang M., Zhang L., Zhu W., Zhang J., Kim S.H., Wang Y., Ni L., Telljohann R., Monticone R.E., McGraw K. (2018). Calorie restriction curbs proinflammation that accompanies arterial aging, preserving a youthful phenotype. J. Am. Heart Assoc..

[18] Wang M., Jiang L., Monticone R.E., Lakatta E.G. (2014). Proinflammation: the key to arterial aging. Trends Endocrinol. Metab..

[19] Chung H.Y., Kim D.H., Lee E.K., Chung K.W., Chung S., Lee B., Seo A.Y., Chung J.H., Jung Y.S., Im E. (2019). Redefining chronic inflammation in aging and age-related diseases: proposal of the senoinflammation concept. Aging Dis..

[20] Lu B., Nakamura T., Inouye K., Li J., Tang Y., Lundbäck P., Valdes-Ferrer S.I., Olofsson P.S., Kalb T., Roth J. (2012). Novel role of PKR in inflammasome activation and HMGB1 release. Nature.

[21] Hu J.J., Liu X., Xia S., Zhang Z., Zhang Y., Zhao J., Ruan J., Luo X., Lou X., Bai Y. (2020). FDA-approved disulfiram inhibits pyroptosis by blocking gasdermin D pore formation. Nat. Immunol..

[22] Kim Y., Park J., Kim S., Kim M., Kang M.G., Kwak C., Kang M., Kim B., Rhee H.W., Kim V.N. (2018). PKR senses nuclear and mitochondrial signals by interacting with endogenous double-stranded RNAs. Mol. Cell.

[23] Guzik T.J., Cosentino F. (2018). Epigenetics and immunometabolism in diabetes and aging. Antioxid. Redox Signal..

[24] Newton K., Dixit V.M., Kayagaki N. (2021). Dying cells fan the flames of inflammation. Science.

[25] Kayagaki N., Kornfeld O.S., Lee B.L., Stowe I.B., O'Rourke K., Li Q., Sandoval W., Yan D., Kang J., Xu M. (2021). NINJ1 mediates plasma membrane rupture during lytic cell death. Nature.

[26] Yang X., Cheng X., Tang Y., Qiu X., Wang Y., Kang H., Wu J., Wang Z., Liu Y., Chen F. (2019). Bacterial endotoxin activates the coagulation cascade through gasdermin D-dependent phosphatidylserine exposure. Immunity.

[27] Abraham N., Stojdl D.F., Duncan P.I., Méthot N., Ishii T., Dubé M., Vanderhyden B.C., Atkins H.L., Gray D.A., McBurney M.W. (1999). Characterization of transgenic mice with targeted disruption of the catalytic domain of the double-stranded RNA-dependent protein kinase, PKR. J. Biol. Chem..

